# Increase of CD4^+^CD25^high^FoxP3^+^ cells impairs *in vitro* human microbicidal activity against *Mycobacterium tuberculosis* during latent and acute pulmonary tuberculosis

**DOI:** 10.1371/journal.pntd.0009605

**Published:** 2021-07-29

**Authors:** Lorenzzo Lyrio Stringari, Luciana Polaco Covre, Flávia Dias Coelho da Silva, Vivian Leite de Oliveira, Maria Carolina Campana, David Jamil Hadad, Moisés Palaci, Padmini Salgame, Reynaldo Dietze, Daniel Cláudio de Oliveira Gomes, Rodrigo Ribeiro-Rodrigues

**Affiliations:** 1 Núcleo de Doenças Infecciosas, Universidade Federal do Espírito Santo, Vitória, Brazil; 2 Center for Emerging Pathogens, Rutgers-New Jersey Medical School, International Center for Public Health, Newark, New Jersey, United States of America; 3 Global Health & Tropical Medicine, Instituto de Higiene e Medicina Tropical, Universidade Nova de Lisboa, Lisbon, Portugal; 4 Núcleo de Biotecnologia, Universidade Federal do Espírito Santo, Vitória, Brazil; Universite Libre de Bruxelles Faculte de Medecine, BELGIUM

## Abstract

**Background:**

Regulatory T cells (Tregs) play a critical role during *Mycobacterium tuberculosis* (*Mtb*) infection, modulating host responses while neutralizing excessive inflammation. However, their impact on regulating host protective immunity is not completely understood. Here, we demonstrate that Treg cells abrogate the *in vitro* microbicidal activity against *Mtb*.

**Methods:**

We evaluated the *in vitro* microbicidal activity of peripheral blood mononuclear cells (PBMCs) from patients with active tuberculosis (TB), individuals with latent tuberculosis infection (LTBI, TST+/IGRA+) and healthy control (HC, TST-/IGRA-) volunteers. PBMCs, depleted or not of CD4^+^CD25^+^ T-cells, were analyzed to determine frequency and influence on microbicidal activity during *in vitro Mtb* infection with four clinical isolates (S1, S5, R3, and R6) and one reference strain (H37Rv).

**Results:**

The frequency of CD4^+^CD25^high^FoxP3^+^ cells were significantly higher in *Mtb* infected whole blood cultures from both TB patients and LTBI individuals when compared to HC. Data from CD4^+^CD25^+^ T-cells depletion demonstrate that increase of CD4^+^CD25^high^FoxP3^+^ is associated with an impairment of Th-1 responses and a diminished *in vitro* microbicidal activity of LTBI and TB groups.

**Conclusions:**

Tregs restrict host anti-mycobacterial immunity during active disease and latent infection and thereby may contribute to both disease progression and pathogen persistence.

## Author summary

Our immune system has an enormous capacity of recognizing and responding to foreign antigens and, likewise, presents an extremely efficient mechanism of controlling these responses. Here, we investigated how a specific cell type with regulatory abilities can interfere in the immunological response against tuberculosis bacillus. For this, we used blood samples from individuals sensitized with the bacillus and patients with active pulmonary tuberculosis to understand how these cells act and their impact on the host/parasite relationship in the development of the disease. We could observe the negative impact that such regulatory cells cause during the immune response against *Mycobacterium tuberculosis*, decreasing the control/elimination of the bacillus in asymptomatic individuals and patients with tuberculosis. We also observed a recovery in the immune response when Treg cells were removed during *in vitro* challenge, restoring the capacity of Mtb clearance. Thus, these regulatory cells, when present, may represent a possible facilitator of the asymptomatic permanence of the bacillus, or even of the development of the disease itself. These data allowed us to see latency and tuberculosis from a new angle and thus postulate new approaches to fight tuberculosis.

## Introduction

Although it has been estimated that one-fourth of the world’s population is latently infected with *Mycobacterium tuberculosis* (*Mtb*), 90% of these individuals may never develop active disease, suggesting that, in most cases, the immune response can control the infection [1,2]. However, despite its ability to control the infection, host immunity is not capable of eliminating Mtb bacilli and cumulative data suggest that T regulatory (Treg) cells in tuberculosis (TB) may play a negative role in *Mtb* clearance [3,4]. Human Treg cells represent 5–10% of CD4^+^ T cell population and can be characterized by the expression of Forkhead box P3 transcription factor (FoxP3) [5]. These cells are known to down-regulate the activity of CD4^+^ and CD8^+^ T-cell populations, avoiding collateral-tissue damage due to an excessive inflammation elicited during the response to pathogens [6,7]. Although this mechanism is generally beneficial to the host in the acute phase, it may be detrimental during chronic infection, when, despite the presence of active immune response, pathogen persistence is sustained, as reported during TB [8].

Tuberculosis patients present elevated frequencies of induced Treg cells when compared to healthy individuals [3,9–14]. Higher level of Tregs, as well as activated CD4^+^ T cells, was also determined in lymph nodes (LN) mononuclear cells of patients with LN TB when compared to PBMCs [15]. Moreover, in TB patients, Treg cells have a pivotal role in depressing anti-Mtb immunity, decreasing IFN-γ production, which is restored after Treg depletion [13,16,17]. Interestingly, the high frequency of Treg population is not restricted to active TB disease, but also reported during latent TB infection (LTBI) when compared to healthy controls (HC) both in PBMC [14,18,19] or in bronchoalveolar lavage [19].

It has been suggested that patients who had been treated and cured for TB present a higher risk of developing pulmonary TB if re-infected [20]. Besides, TB-case contacts with a prior positive TST response had an increased propensity to develop the active disease when compared to individuals with a negative TST result [21,22]. Recently our group showed that TST-positive individuals had an impaired in vitro microbicidal activity compared to TST-negative [23]. Together these findings indicate that immune-competent individuals infected with *Mtb* are not protected against disease during subsequent reinfection [24–26].

In the present work, we investigated the ability of CD4^+^CD25^high^FoxP3^+^ cells to down-modulate *in vitro* microbicidal activity against *Mtb*. Data presented here demonstrate that Treg cells hinder anti-*Mtb in vitro* microbicidal activity, suggesting that these cells may inhibit the establishment of protective immunity against *Mtb*, contributing to pathogen persistence and/or disease progression in LTBI individuals and patients with active TB.

## Materials and methods

### Ethics statement

All study participants signed an informed consent form before their enrolment and before sample collection. The study protocol was approved by the Health Science Center Internal Review Board of the Universidade Federal do Espírito Santo and is registered under the number CEP/UFES 080/2011.

### Human subjects

HIV-negative healthy volunteers were invited to participate in the present study at Universidade Federal do Espírito Santo (UFES), Vitória, Brazil, and divided according to their purified protein derivative (TST) and whole blood IFN-γ release assay (IGRA) status in LTBI (TST+/IGRA+) and healthy control (TST-/IGRA-) groups. Both LTBI and HC subjects underwent a comprehensive clinical evaluation before their enrollment, which included a complete hemogram, HIV test, thoracic X-ray, TST, and IGRA testing. Candidates invited to participate in the present study were excluded if positive for other chronic diseases such as HIV, intestinal helminths, hepatitis, and autoimmune diseases. HIV-negative active pulmonary TB patients were enrolled at the Tuberculosis Clinic, Hospital Universitário Cassiano Antônio de Moraes, Centro de Ciências da Saúde, Universidade Federal do Espírito Santo. TB diagnosis was based on chest X-ray, acid-fast bacilli staining of sputum smears (AFB smears), and mycobacterial culture of sputum samples from all TB patients. Blood samples from TB patients were collected before anti-TB therapy was initiated. Enrolled subjects/patients, who initiated anti-TB treatment, or the use of anti-inflammatory drugs were also excluded.

### Tuberculin skin test (TST)

TST involved an intradermal injection of 0.1mL (2 tuberculin units) of the purified protein derivative RT23 (Statens Serum Institut, Copenhagen, Denmark) into the anterior surface of the forearm with a standard tuberculin syringe. Reactions were evaluated 72h after the injection by two experts and defined as positive by induration of ≥10 mm in the transverse diameter using the “Sokal ballpoint pen method” [27].

### QuantiFERON TB-Gold In-Tube (QFT-IT)

Prior to the intradermal injection of PPD (tuberculin skin testing), blood samples for IGRA testing were collected by venipuncture using vials provided by the manufacturer. Blood samples were used for whole blood IFN-γ release assay (QuantiFERON TB Gold In-Tube, Qiagen, Valencia, CA, USA) according to the manufacturer’s instructions.

### Mycobacterium tuberculosis reference strain and clinical isolates

The ATCC *Mtb* H37Rv reference strain (ATCC27294) and four clinical isolates with distinct minimal inhibitory concentration (MIC) to first-line anti-Mtb drugs and IS6110 RFLP electrophoretic band pattern [28], stored at the Núcleo de Doenças Infecciosas (NDI)/UFES repository, were used in the present study **(S1 Fig and Table 1).**

**Table 1:** Minimal inhibitory concentration (MIC) of *Mycobacterium tuberculosis* clinical isolates used in the present study.

Isolate Id	Specimen	INH	RMP	PZA	EMB	SM
**S1**	Sputum	<0,1	<2,0	<100,0	<2,5	<2,0
**S5**	Sputum	<0,1	<2,0	<100,0	<2,5	<2,0
**R3**	Sputum	4,0	32,0	>900,0	<2,5	>128,0
**R6**	Sputum	>4,0	>32,0	<100,0	<2,5	<2,0

**MIC**: Minimal inhibitory concentration; **INH**: Isoniazid; **RMP**: Rifampicin; **PZA**: Pyrazinamide; **EBM**: Ethambutol; **SM**: Streptomycin.

### Cell cultures and in vitro microbicidal activity assay (Mtb killing assay)

Peripheral blood samples were collected by venipuncture in K_3_-EDTA-treated tubes (BD Vacutainer Blood Collection Tube, Becton & Dickinson, USA). Peripheral blood mononuclear cells (PBMC) were isolated by density gradient separation (Ficoll-Histopaque, SIGMA-ALDRICH, Missouri, USA). The *in vitro* microbicidal activity assay, a modified version of an *ex-vivo* model previously described [29], was performed using 300 μL of heparinized peripheral whole blood or PBMC, depleted or not of Treg cells, dispensed into sterile microtubes (Sarstedt AG & Co, Nümbrecht, Germany) and incubated at 37°C, 5% CO_2_ in the presence of Mtb suspension (10:1 MOI) for 72h. Non-stimulated control samples received complete RPMI medium alone. After incubation, cultures were centrifuged (15000 x *g* for 10 min) and the supernatants were collected and stored at -80°C until further use. The cell pellet was lysed in sterile Milli-Q water, followed by serial dilution (10^−1^ to 10^−4^) in PBS 0.25% Tween 80. Aliquots were plated on Middlebrook 7H11 medium (BD Difco, Detroit, Michigan, USA) supplemented with Middlebrook OADC (BD BBL, Maryland, USA) and incubated for 14–21 days at 37°C in 5% CO_2_ when colony forming units (CFU) was determined. All experiments were conducted in triplicates.

### Depletion of CD4^+^CD25^+^ regulatory T cells

Depletion of Treg cells was accomplished by magnetic separation using MACS human CD4 CD25 regulatory T cell isolation kit according to manufacturer**’**s instructions (Miltenyi Biotec, Germany). Briefly, CD4^+^ cells were first isolated by negative selection, then CD4^+^CD25^+^ cells were isolated by positive selection, resulting in 95% purity based on FoxP3 expression as reported previously [13] **(S2A and S2B Fig).**

### Flow cytometry

Intra- and extracellular labeling were performed using monoclonal antibodies (MoAb) (BD Immunocytometry, BD Biosciences, San Jose, CA, USA; Biolegend, California, USA) conjugated with fluorescein isothiocyanate (FITC); phycoerythrin (PE), peridinin chlorophyll (PerCP), phycoerythrin-cyanine 7 (PE-Cy7). Cell surface markers were labeled using MoAb specific for CD25 (clone BC96), CD4 (SK3) markers, isotype control IgG2a (eBM2a), IgG2b (eBMG2b) and anti-IgG1 (P3). Cell surface markers were first labeled in FACS buffer (PBS 0.1% BSA 0.05% sodium azide) at 4°C for 30 min. Then, cells were washed, fixed, permeabilized using FoxP3 Staining Buffer (eBioscience, CA, USA), and incubated at 4°C for 30 min. for intracellular labeling of T-bet (a Th1 specific T-box transcription factor expressed in T cell) (clone 4B10) and FoxP3 (Forkhead box P3 transcription factor) (clone 236A/E7). Data was acquired with Attune NxT flow cytometer (Life Technologies, Carlsbad, CA, USA) and analyzed using FlowJo software (TreeStar, San Carlos, CA, USA).

### Detection of Soluble IFN-γ

Soluble IFN-γ concentration in culture supernatants was measured using a commercial kit according to the manufacturer’s instructions (Human ELISA Ready-Set-Go kit; eBioscience Inc. California, San Diego, USA). The absorbance was recorded at 570 nm and 450 nm on SpectraMax M3 (Molecular Devices, Sunnyvale, CA, USA), and the detection limit was 4pg to IFN-γ.

### *In vitro* evaluation of phagocytic activity in differentiated human macrophages

PBMCs (1×10^6^ cells/mL) were incubated at 37°C/5%CO_2_ in 16-well tissue culture chamber slides (Nalge Nunc International). Four hours after incubation, monolayers were washed three times with RPMI 1640 medium supplemented with 10% inactivated fetal bovine serum (FBS) to remove non-adherent cells. Adherent cells were cultivated with rGM-CSF (10μg/mL stock R&D) for 5 days, followed by infection with either H37Rv strain or R6 Mtb isolate (MOI of 10:1) for 4h, then washed three times with 10% FBS RPMI 1640. Cells were divided in two samples: one readily fixed and stained using the Kinyoun method as described previously [30], and another group incubated at 37°C for 24h, followed by staining. Slides were examined using an optical microscope (Zeiss Axiostar) and intracellular bacteria load counted (number of cells containing intracellular bacilli x 200 fields).

### Statistical analyses

Normally distributed variables were compared using ANOVA analysis simple factorial test and by one-way ANOVA-Tukey’s honestly significant difference (Tukey’s HSD) post-hoc method. A 5% significance level (p<0.05) was used to determine statistical significance. Statistical analysis was performed using GraphPad Prism software 7.0 (GraphPad Software, San Diego, CA, EUA).

## Results

### Characteristics of the studied population

Thirty-seven HIV-negative subjects were enrolled and divided in three groups: a) Healthy Controls (n = 13), b) LTBI (n = 13), and c) TB group (n = 11). Healthy control group comprised 3 males (median age ± SD = 31±10.6 years) and 10 females (26.5±5.1 years), the LTBI group 4 males (29±6.2 years) and 9 females (30±9.8 years) and the TB group 6 males (39±8.6 years) and 5 females (28±6.6 years), as described in Table 2. BCG vaccination after birth is mandatory in Brazil since 1960; therefore, all participants were vaccinated before enrollment. Age and gender distribution did not differ significantly among the studied groups, patients with helminthiasis were excluded. A few individuals with a discordant result between TST and IGRA (TST-/IGRA+ or TST+/IGRA-) were excluded to prevent bias during further analyzes.

**Table 2:** Description of all participants of the study.

Study Demographics	Healthy Control	LTBI	*Patient (Active Pulmonary Tuberculosis)*
**Sample Size**	13	13	11
**Gender**	10 (77%) Females	9 (70%) Females	5 (45%) Females
	3 (23%) Males	4 (30%) Males	6 (55%) Males
**Age**	28.23 (±7.49)	34 (±9.33)	38.55(±2.77)
**Helminth infection**	0%	0%	36%^A^
**BCG vaccination status**	13/13 (100%)	13/13 (100%)	11/11 (100%)
**TST**	13/13 (100%)	13/13 (100%)	*NP*
*TST status*	Neg	Pos	*NP*
*TST values*	0	15.23 (±2.77)	*NP*
**IGRA**	13/13 (100%)	13/13 (100%)	*NP*
*IGRA status*	Neg	Pos	*NP*
*TB Ag-NIL values*	0.01 (±0.04)	4.45 (±4.61)	*NP*
*MIT-NIL values*	15.92 (±8.4)	8.66 (±4.32)	*NP*
**Sputum collection**	*NP*	13/13 (100%)	11/11 (100%)
Baciloscopy	*NP*	Neg	3+
Culture	*NP*	Neg	Pos
**Chest Abnormalities (TB)**	No Present	No Present	Present
**HIV**	Neg	Neg	Neg
**Hepatitis**	Neg	Neg	Neg

Data are presented as mean ± SD, n (%). **IGRA:** Interferon-γ release assay; **TB:** tuberculosis; **BCG:** Bacille Calmette–Guérin; **TST:** tuberculin skin test; **NP:** Not performed**; Pos:** Positive**; Neg;** Negative; **A) Helminth found: 2 females with** Strongyloides stercoralis and Schistosoma mansoni; 2 males with Trichiura trichuris, and Necator americanus.

### Ex-vivo frequency of CD4^+^CD25^high^FoxP3^+^ cells are high in TB patients and LTBI individuals when compared to healthy controls

**Fig 1:**
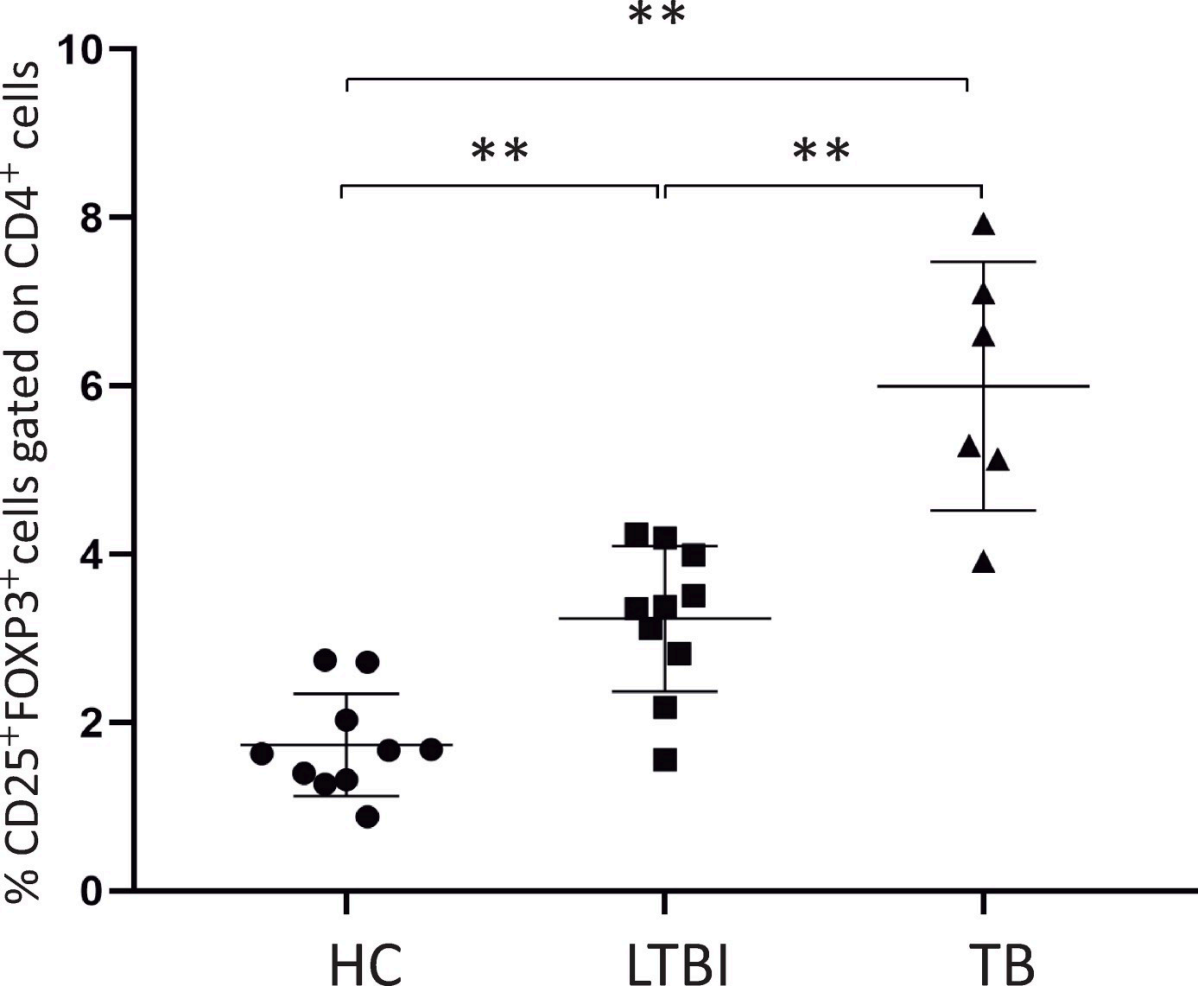
*Ex-vivo f*requency of CD4^+^CD25^high^FoxP3^+^ in TB patients are higher when compared to HC and LTBI subjects. Peripheral blood from healthy control subjects (HC, n = 10), latent infected individuals (LTBI, n = 10), and active pulmonary TB patients (TB, n = 6). *Ex vivo* samples were collected and immunophenotyped for the presence of CD4^+^CD25^high^FoxP3^+^ cells (** = p<0.01).

After venipuncture, whole blood samples were immediately stained for CD4^+^CD25^high^FoxP3^+^ and analyzed. A high frequency of CD4^+^CD25^high^FoxP3^+^ cells was observed in patients with active pulmonary tuberculosis when compared to healthy control and LTBI subjects, and when LTBI patients were compared to healthy controls (p<0.05) (Fig 1).

### Frequency of CD4^+^CD25^high^FoxP3^+^ cells in LTBI individuals and TB patients is significantly augmented in whole blood cultures after Mtb infection

**Fig 2:**
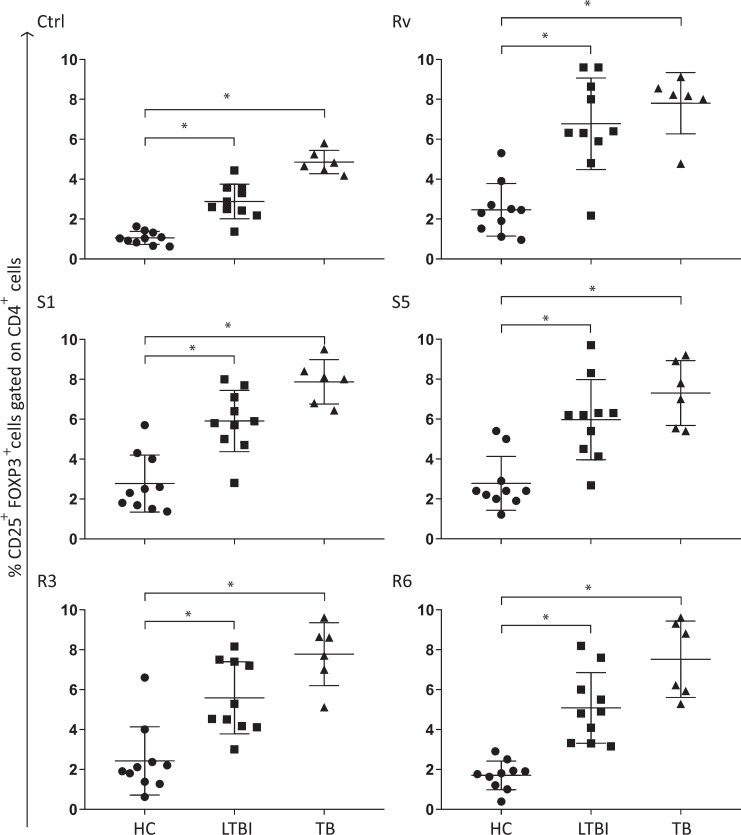
Frequency of CD4^+^CD25^high^FoxP3^+^ cells is increased in peripheral blood of LTBI and active TB patients. Peripheral blood from healthy control subjects (HC, n = 10), latent infected individuals (LTBI, n = 10), and active pulmonary TB patients (TB, n = 6) were cultivated in the presence or absence of live *Mtb* by 72 hours, followed by staining with fluorochrome-conjugated antibodies to CD4, CD25 and FoxP3 and flow cytometry analysis. Frequency of CD4^+^CD25^high^FoxP3^+^ cells was determined after *in vitro* infection with multidrug sensitive (S1 and S5) clinical isolates, multidrug-resistant (R3 and R6) and reference strain H37RV. Statistical relevance was assessed using the ANOVA test. Bars indicating statistical significance between groups are shown (* = p<0.05); ***Ctrl =*** *Control*.

To investigate if CD4^+^CD25^high^FoxP3^+^ cell frequency differed among the studied groups, whole peripheral blood samples from TB patients, healthy control and LTBI subjects were cultured for 72h in the presence or absence of live *Mtb* H37Rv strain. After, *in vitro stimulation*, CD4^+^CD25^high^FoxP3^+^ cell frequency was further increased, in both LTBI and TB groups, upon exposure to live *Mtb* strain (H37Rv), or upon exposure to five different clinical isolates. On the other hand, no significant change was observed in the healthy control group regardless of the stimuli used **(Fig 2).**

To determine if the observed increase on CD4^+^CD25^high^FoxP3^+^ cell frequency was a strain-dependent phenomenon, the experiment was also conducted with both multidrug-resistant (R3 and R6) and drug-susceptible (S1 and S5) clinical isolates. The same profile observed for H37Rv was also reported for all the isolates used, regardless of its drug susceptibility status **(Fig 2)**, suggesting that the expansion on the Treg population was neither strain/isolate dependent. These results concur with the hypothesis that there is an increase in antigen-specific Treg cell frequency in latent and active TB disease.

### Increase in CD4^+^CD25^high^FoxP3^+^ population is accompanied by a decline in microbicidal activity in whole blood cultures from LTBI and TB donors

**Fig 3:**
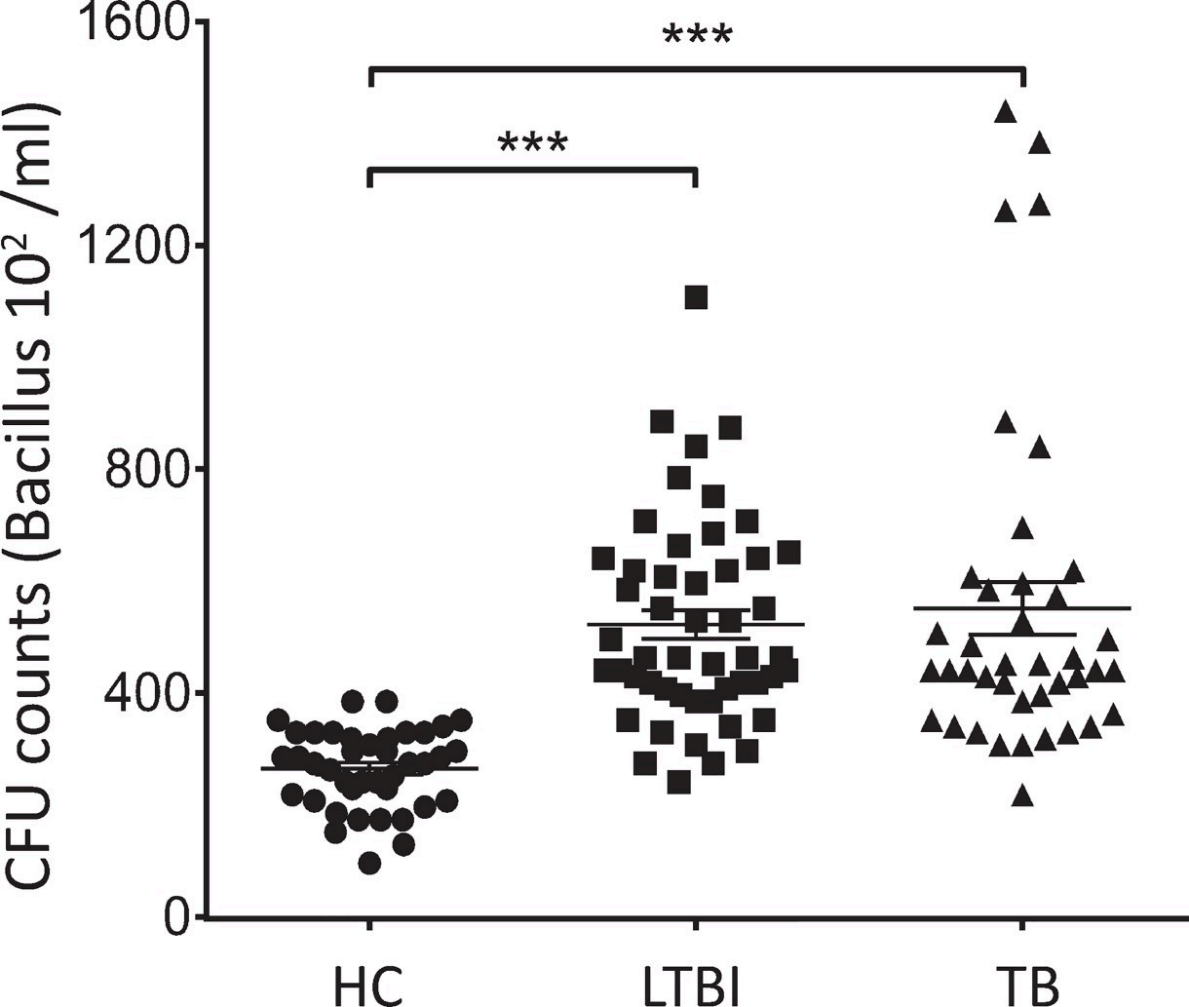
Whole blood microbicidal activity is significantly reduced on both LTBI individuals and active TB patients. Data represent Colony Forming Units (CFU/mL) counts for all the strain and clinical isolates (H37Rv, S1, S5, R3 and R6) in healthy control (n = 10), LTBI (n = 10), and TB (n = 6) groups. Bar indicates the median of CFU/mL counts. Statistical relevance (*** = p< 0.0001) was assessed using ANOVA test. ***CFU =*** *Colony Forming Unit*.

Considering that CD4^+^CD25^high^FoxP3^+^ cell frequency was elevated on both LTBI and TB groups **(Fig 2)**, we analyzed if it could hinder Th1-dependent immune responses and negatively impact *Mtb* clearance. Data presented here show that the increase in CD4^+^CD25^high^FoxP3^+^ cell frequency **(Fig 2)** was accompanied by a significant expansion of CFU/mL numbers, in both LTBI and TB groups, for all the strains/isolates examined **(Fig 3).**

To rule out the possibility that the observed lower CFU/mL counts in the control group was due to impairment of phagocytosis, an *in-vitro* assay to evaluate the phagocytic activity was performed using *in vitro* differentiated macrophages derived from PBMCs of LTBI and healthy controls. As depicted in **S3 Fig**, a similar phagocytic activity was reported in both groups, regardless of incubation time (4 vs 24hs), TST status, or strain/clinical isolate used. These data suggest that microbicidal activity is hindered in LTBI and TB groups and might be associated with the observed Treg cell upregulation.

### Depletion of Treg-like cells restores in vitro microbicidal capacity

To assess whether the reduced microbicidal activity reported for LTBI and TB groups was indeed associated with an increase in CD4^+^CD25^high^FoxP3^+^ cell frequency, *in vitro* microbicidal activity assays using either whole PBMC or Treg-depleted PBMC samples were performed. Before carrying out the depletion experiments, the microbicidal assay using PBMC instead of whole blood samples was validated. PBMC and whole blood samples were obtained from the same donors and processed simultaneously. As demonstrated in **S4 Fig,** results from microbicidal assays using either PBMC or whole blood samples are comparable.

**Fig 4:**
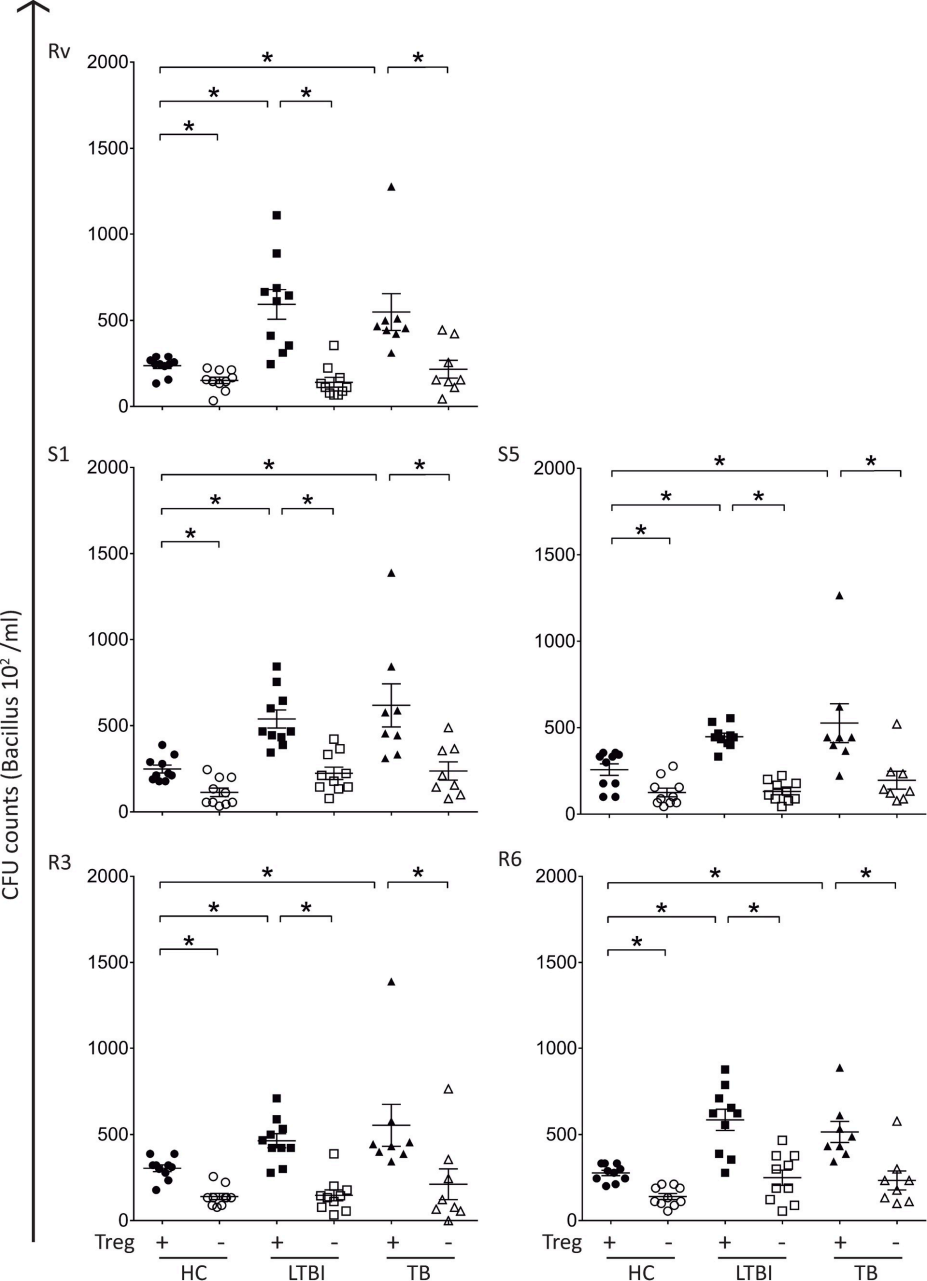
Depletion of CD4^+^CD25^+^ T cells restores *in-vitro* microbicidal activity in PBMC samples from LTBI individuals and TB patients. PBMC samples from healthy control subjects (HC, n = 10), latent infected individuals (LTBI, n = 10) and active TB patients (TB, n = 8) were depleted of CD4^+^CD25^+^ cells. Whole PBMC and Treg-depleted PBMC samples were infected with *Mtb* strains for 72 hours and then processed for quantification of Colony Forming Units (CFU). Individual data from CFU/mL counts on whole PBMC and CD4^+^CD25^+^ cells depleted PBMC cultures infected with the reference strain H37RV, drug-susceptible (S1 and S5) or multidrug-resistant (R3 and R6) isolates. Bar indicates the median value of CFU/mL counts. Statistical relevance (* = p<0.05) was assessed using the ANOVA test. ***CFU =*** *Colony Forming Unit*.

As hypothesized, depletion of CD4^+^CD25^+^ cells restored the *in vitro* microbicidal activity on PBMC samples from LTBI and TB groups to levels comparable to those observed in healthy control subjects **(Fig 4)**. *In vitro* microbicidal activity on both LTBI and TB groups were significantly enhanced after CD4^+^CD25^+^ cell depletion (p*<0*.*05)*, as demonstrated by a strong decline in CFU/mL counts **(Fig 4)**. As observed previously, results were similar regardless of the isolate/strain used **(Fig 4),** confirming that the depletion of these cells boosted the microbicidal activity in PBMC samples from both LTBI and TB groups. Together, these results support our hypothesis that the expansion of Treg cells observed during LTBI, and active TB disease is associated with an impairment of host’s microbicidal activity.

### Depletion of CD4^+^CD25^+^FoxP3^+^ cells restored Th1 profile in LTBI subjects

**Fig 5:**
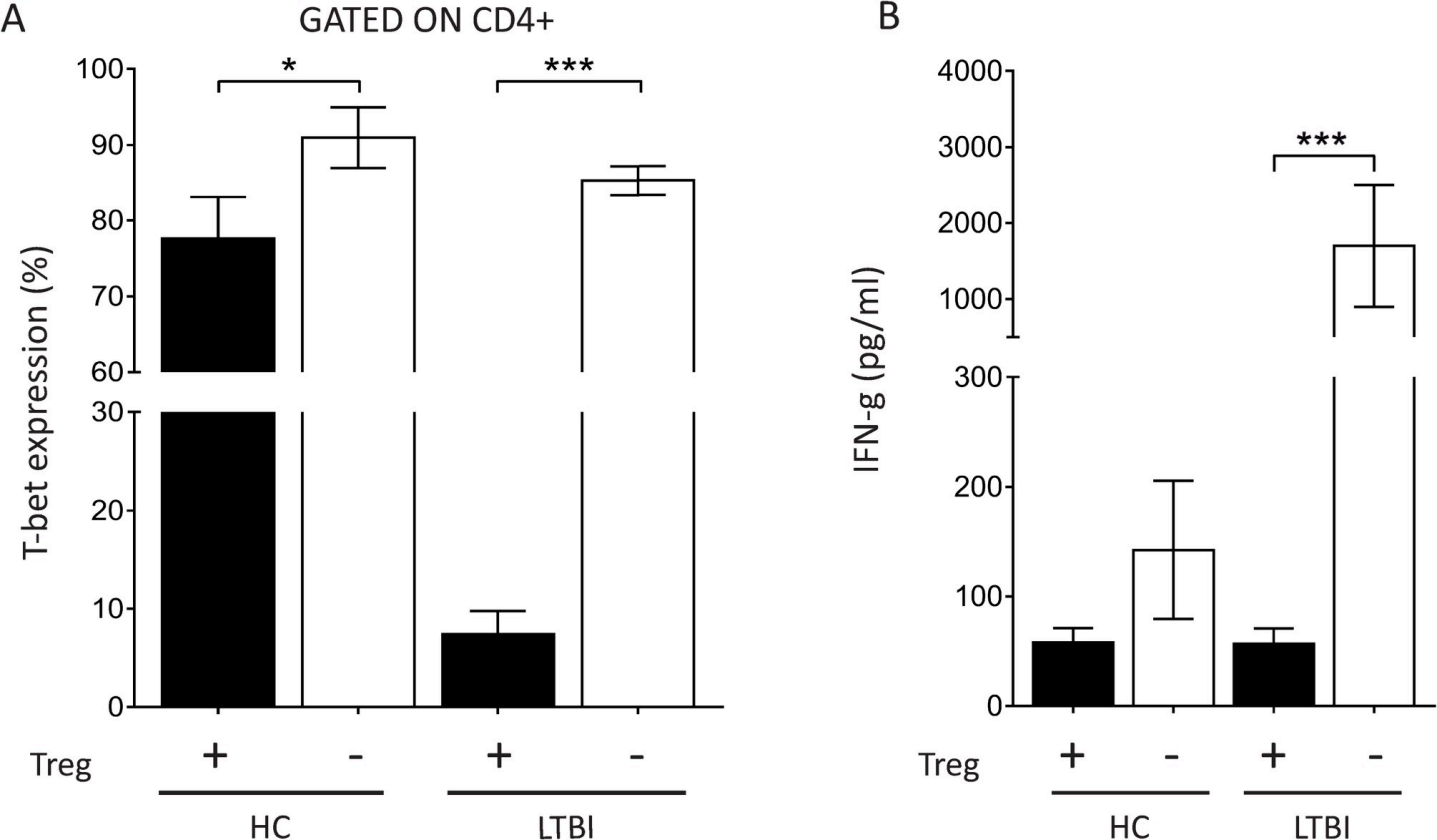
Depletion of CD4^+^CD25^+^FoxP3^+^ T cells restored the TH1 profile in LTBI individuals. PBMC samples from healthy control subjects (HC, n = 5) and latent infected individuals (LTBI, n = 5) were depleted of CD4^+^CD25^+^ cells. Whole PBMC and Treg-depleted PBMC samples were infected with *Mtb* H37Rv strain for 72 hours *in vitro*. (**A)** Intracellular expression of T-bet analyzed on CD4-gated population by flow cytometry; **(B)** Soluble levels of IFN-y in PBMC culture supernatants determined by ELISA. Statistical relevance (* = p<0.05; *** = p<0,0001) was assessed using ANOVA test.

Treg cell expansion has been associated with hindered Th1-dependent immune responses [13]. Therefore, we investigated if intracellular expression of T-bet and secretion of soluble IFN-γ in H37RV infected PBMC cultures were altered in presence or absence of Treg cells. In the presence of Tregs, T-bet expression in healthy controls was close to eight-fold greater than in LTBI subjects. As expected, following Treg depletion, intracellular expression of T-bet on CD4^+^ cells was significantly increased in both groups (**Fig 5A**), with a remarkable intensification in the LTBI group. After Treg depletion, the expression of intracellular T-bet was similar in both control and LTBI groups **(Fig 5A).**

As depicted in **Fig 5B,** the concentration of soluble IFN-γ in PBMC culture supernatants in the LTBI group was boosted significantly after Treg depletion, which corroborates with the T-bet data and the hypothesis that the increase of CD4^+^CD25^high^FoxP3^+^ cells hinders Th1 response.

## Discussion

Progress to TB disease following *Mtb* infection depends on the outcome of a subtle balance between genetic factors involved in susceptibility and resistance [31] and the ability of the host to mount an effective immune response [32–34]. Immune regulatory mechanisms, such as Treg cells, are known to limit inflammatory immune responses [8,35,36] and have been associated with thwarted immune responses against *Mtb* which if overrepresented, may contribute to the persistence and/or establishment of chronic infections [1,4]. Therefore, it is plausible to expect that upregulation in the Treg cell population may lead to disease reactivation [37] or re-infection [13,18,35,38,39]. Data presented here demonstrate that *ex-vivo* (Fig 1) and *in vitro* (Fig 2) Treg population is significantly augmented in LTBI subjects and patients with active TB when compared to healthy controls. These findings are supported by previous studies showing a higher frequency of CD4^+^CD25^high^FoxP3^+^ cells in both pulmonary TB patients and LTBI subjects, when compared to other groups, suggesting a potential role of these cells in disease progression [3,10,11,13,14,16–18,35,40]. Although it is still not clear whether increased *in vitro* Treg cell frequency correlates with progression of TB disease in humans, data exhibiting a significant *in vitro* expansion of these cells on samples from TB patients after stimulation [11,13,36,37,41–43] support this hypothesis. A possible explanation for the observed significant increase in Treg cell frequency in LTBI individuals comes from data showing that CD4^+^ T cells expansion during exposure to *Mtb* could induce the upregulation of Treg cells specific to *Mtb* antigens [13,44].

Other studies suggest that different *Mtb* strains induced Foxp3^+^ cells expansion distinctively [45,46]. Data presented here demonstrated that the observed Treg frequencies associated with drug-susceptible (DS strains, H37Rv, S1 and S5) and drug-resistant (DR strains, R3 and R6) *Mtb* strains in whole blood cultures did not differ **(Fig 2)**, concurring with Lim et al. [47]. These authors showed that frequencies of Treg cells in TB patients infected with either drug-susceptible Mtb or multidrug-resistant *Mtb* were similar but significantly higher when compared to healthy controls [47], corroborating with our findings.

A modified *ex vivo* whole blood bactericidal assay [29] allowed us to confirm that the upregulation of CD4^+^CD25^high^FoxP3^+^ cells was associated with impaired microbicidal activity in latent and active TB groups when compared to healthy control subjects, regardless of the strain used. This difference was not due to alterations in bacterial phagocytosis assay **(S3 Fig)** but the depletion of CD4^+^CD25^high^FoxP3^+^ cells in the culture restored the microbicidal activity in LTBI and TB group regardless of Mtb strain/isolates used. This result corroborates previous findings showing that peripheral blood Tregs from patients with pulmonary TB hindered the ability of alveolar and monocyte-derived macrophages to restrict *Mtb* growth [48]. Data presented here also confirm previous results showing that CD4^+^CD25^high^FoxP3^+^ cells depletion in PBMC cultures from LTBI subjects restores IFN-γ production and expression of T-bet, enhancing the Th1-immune response **(Fig 5A and 5B)** [12,13].

Our work has limitations. First, since a large proportion of patients with pulmonary tuberculosis were under the use of anti-inflammatory drugs, which could hinder the assessment of cytokines, cell phenotype, and the “in vitro” killing assay; enrolling TB patients who were not using such medications to participate in the present study was a challenge, which limited the number of enrolled TB patients. Second, frequently the final volume of collected blood samples was smaller than required, impacting directly on the number of experiments that could be performed per subject. Third, the great majority of enrolled TB patients refused to provide stool samples for intestinal worms testing; therefore, the impact of the presence of intestinal worms could not be assessed. Fourth, all enrolled patients with pulmonary tuberculosis presented a 3+ or higher grade bacilloscopy result, which hindered the correlation of bacillary load and the “in vitro” killing assay results. And finally, we were unable to enroll discordant cases separately, which due to the necessary sampling size would require mass testing for both IGRA and TST; therefore, analyzing discordant patients as a separate group was not possible. Limitations, notwithstanding, in this study, we demonstrate that an increase in CD4^+^CD25^high^FoxP3^+^ population thwarts the host´s ability to restrict Mtb growth. Therefore, it may facilitate both mycobacterial survival and intracellular persistence *in vivo*. Taken together, the results presented here may contribute to further our knowledge about the reason why the age-adjusted incidence rate of TB reinfection after treatment was four times greater than new TB cases [20]. Additionally, it also may help to elucidate why case contacts with prior positive TST and people who had TB previously present a significantly higher risk of developing active TB disease if reinfected, when compared to healthy subjects [21,22]. Our findings support the hypothesis that both latent and active Mtb infection led to an upregulation in Treg cell frequency, which in turn may down-regulate the ability of the host to kill intracellular mycobacteria, significantly increasing the risk of TB disease progression/reactivation in LTBI individuals or of reinfection of those treated and cured after a re-exposure to *Mtb*.

## Supporting information

S1 Fig IS6110 RFLP pattern for Mtb clinical isolates used in the study. RFLP patterns demonstrate that selected clinical isolates are distinct from each other. Legend: S1 and S5 –drug-susceptible isolates, R3 and R6—drug-resistant isolates, and CR–MT 14323 reference strain used for IS6110 RFLP typing.
(TIF)

S2 Fig Gating strategy. **(A)** Specific gating strategy was performed using CD4^+^, CD25^high+^ and FoxP3^+^ expression before and after depletion of CD4^+^CD25^+^ cells. (**B)** FoxP3^+^ expression assessed by flow cytometry analysis of intracellular FOXP3^+^ on CD4^+^ cells before and after CD4^+^CD25^+^ depletion assay (*** = p<0.0001).
(TIF)

S3 Fig Evaluation of *in vitro* phagocytic activity in human differentiated macrophages upon infection with live *M*. *tuberculosis*. Phagocytic activity was evaluated ***in vitro*** in PBMC samples from healthy control subjects (HC) or latently infected individuals (LTBI) by quantification of intracellular mycobacteria after 4h and 24h of exposure to live Mtb H37Rv strain and R6 isolate, via Kinyoun acid-fast staining. Bars indicating statistical significance between groups are shown (* = p<0.05).
(TIF)

S4 Fig Validation of the *in-vitro* microbicidal activity assay using PBMC samples. Paired whole blood (WB) and PBMC samples from healthy control subjects (n = 10), LTBI individuals (n = 10) and TB patients (n = 7) were infected with the reference strain H37RV or drug-susceptible (S1 and S5) or multidrug-resistant (R3 and R6) clinical isolates.
(TIF)
