# Supplementary figures and images for: Increase of CD4+CD25highFoxP3+ cells impairs in vitro human microbicidal activity against Mycobacterium tuberculosis during latent and acute pulmonary tuberculosis
Source: PLoS Negl Trop Dis. 2021 Jul 29;15(7):e0009605. doi: 10.1371/journal.pntd.0009605 (PMC8321116; doi:10.1371/journal.pntd.0009605)

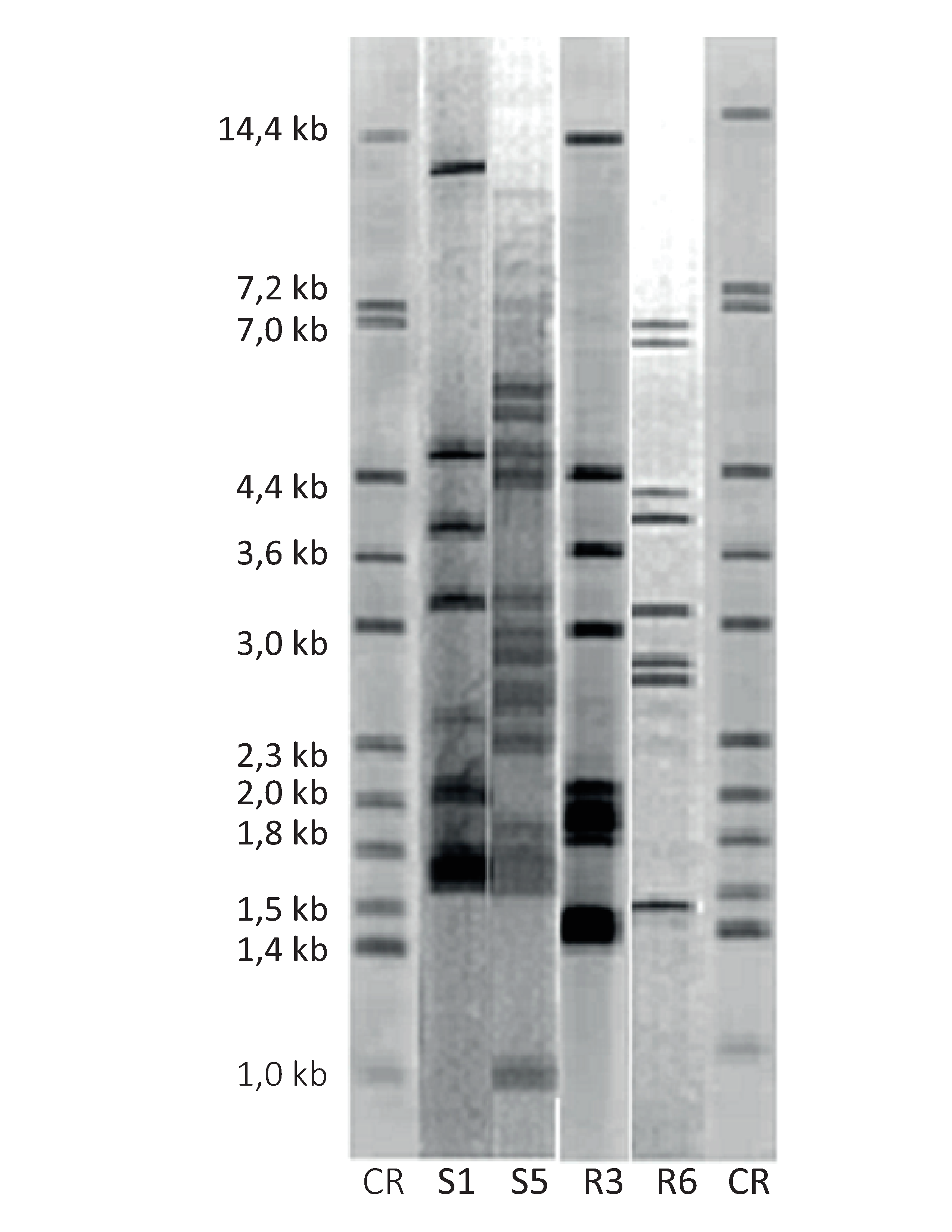

Supplement: S1 Fig — RFLP patterns demonstrate that selected clinical isolates are distinct from each other. Legend: S1 and S5 –drug-susceptible isolates, R3 and R6—drug-resistant isolates, and CR–MT 14323 reference strain used for IS6110 RFLP typing. (TIF) [file pntd.0009605.s001.tif]

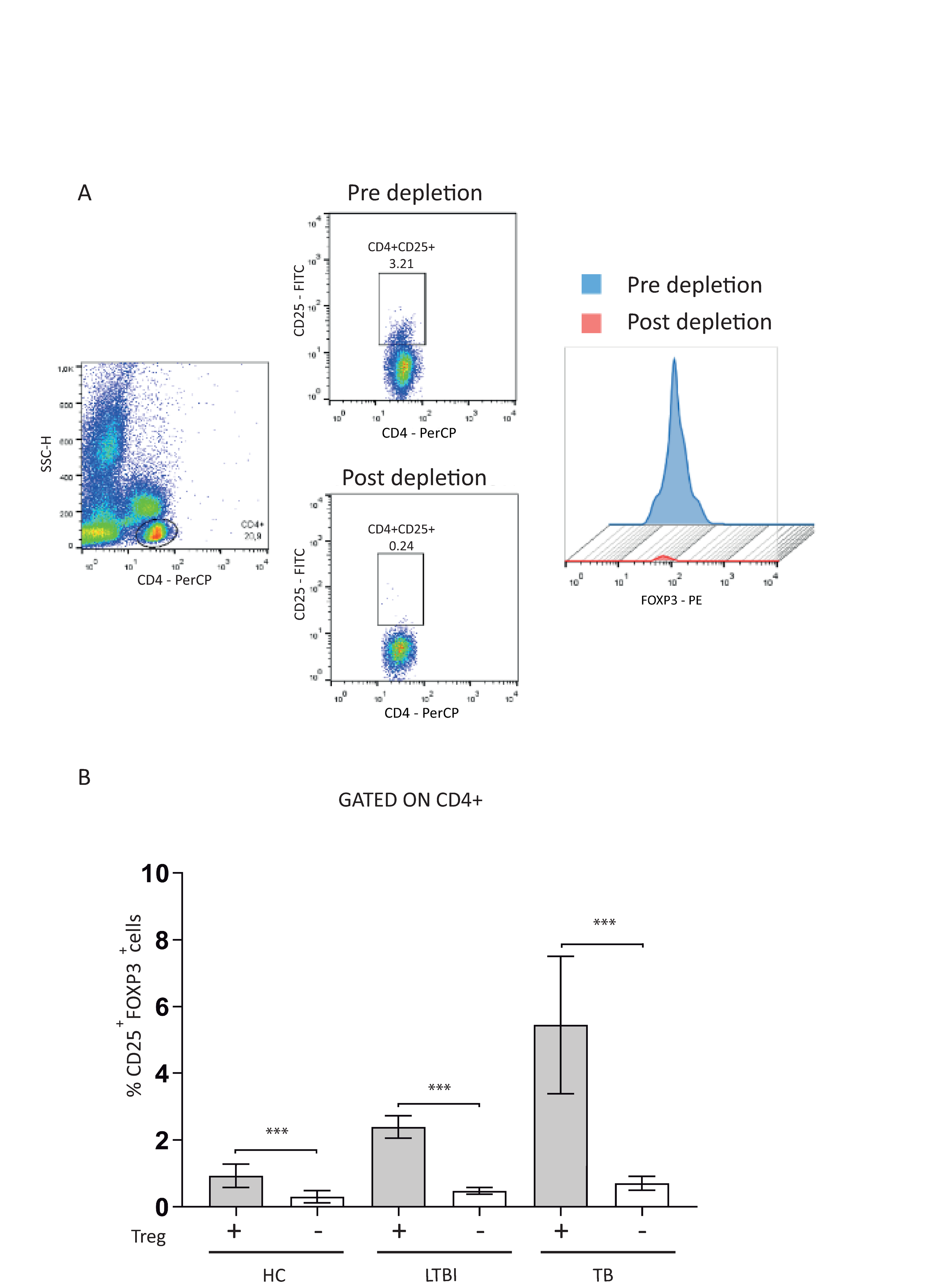

Supplement: S2 Fig — (A) Specific gating strategy was performed using CD4+, CD25high+ and FoxP3+ expression before and after depletion of CD4+CD25+ cells. (B) FoxP3+ expression assessed by flow cytometry analysis of intracellular FOXP3+ on CD4+ cells before and after CD4+CD25+ depletion assay (*** = p<0.0001). (TIF) [file pntd.0009605.s002.tif]

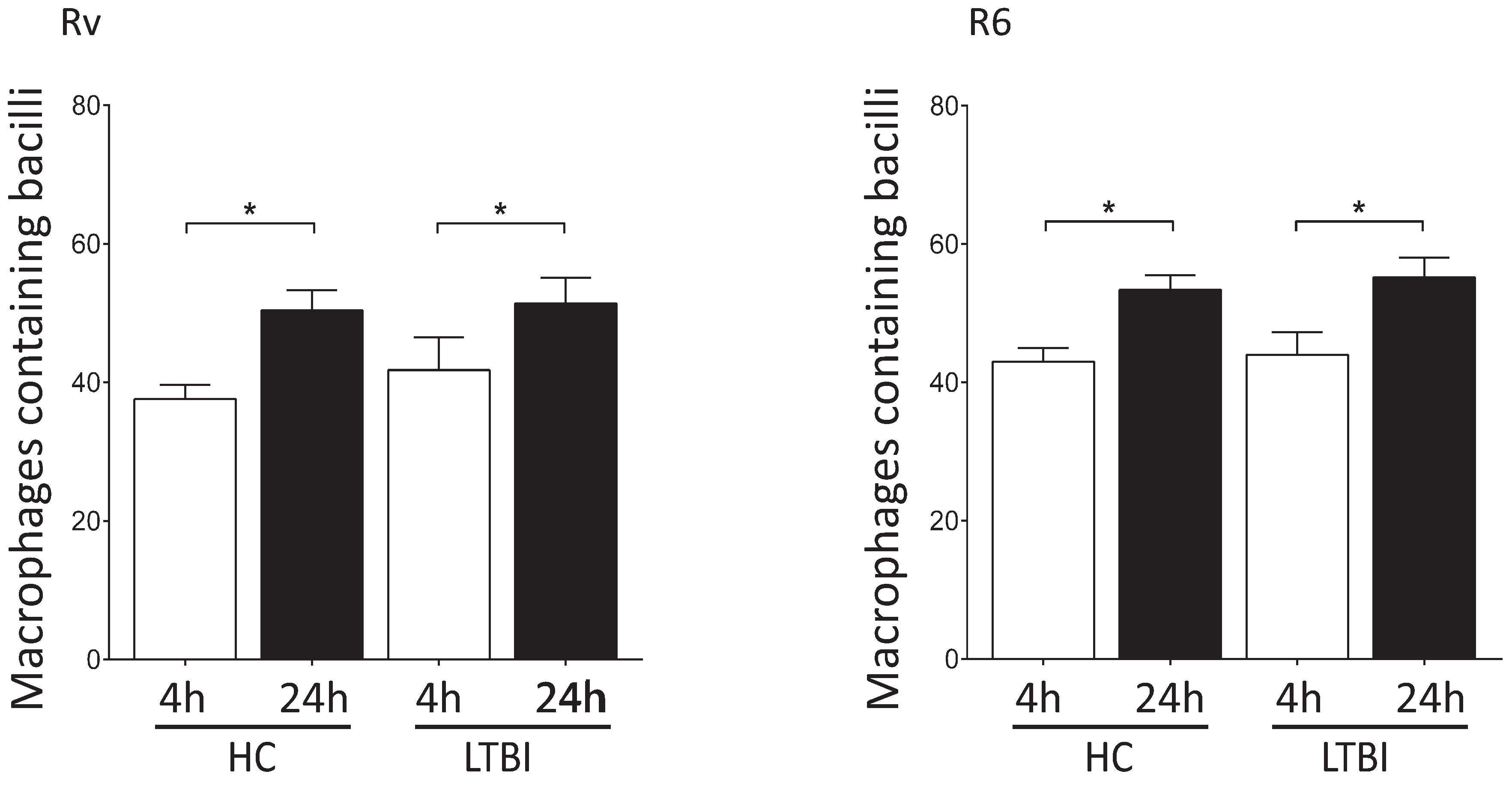

Supplement: S3 Fig — Phagocytic activity was evaluated in vitro in PBMC samples from healthy control subjects (HC) or latently infected individuals (LTBI) by quantification of intracellular mycobacteria after 4h and 24h of exposure to live Mtb H37Rv strain and R6 isolate, via Kinyoun acid-fast staining. Bars indicating statistical significance between groups are shown (* = p<0.05). (TIF) [file pntd.0009605.s003.tif]

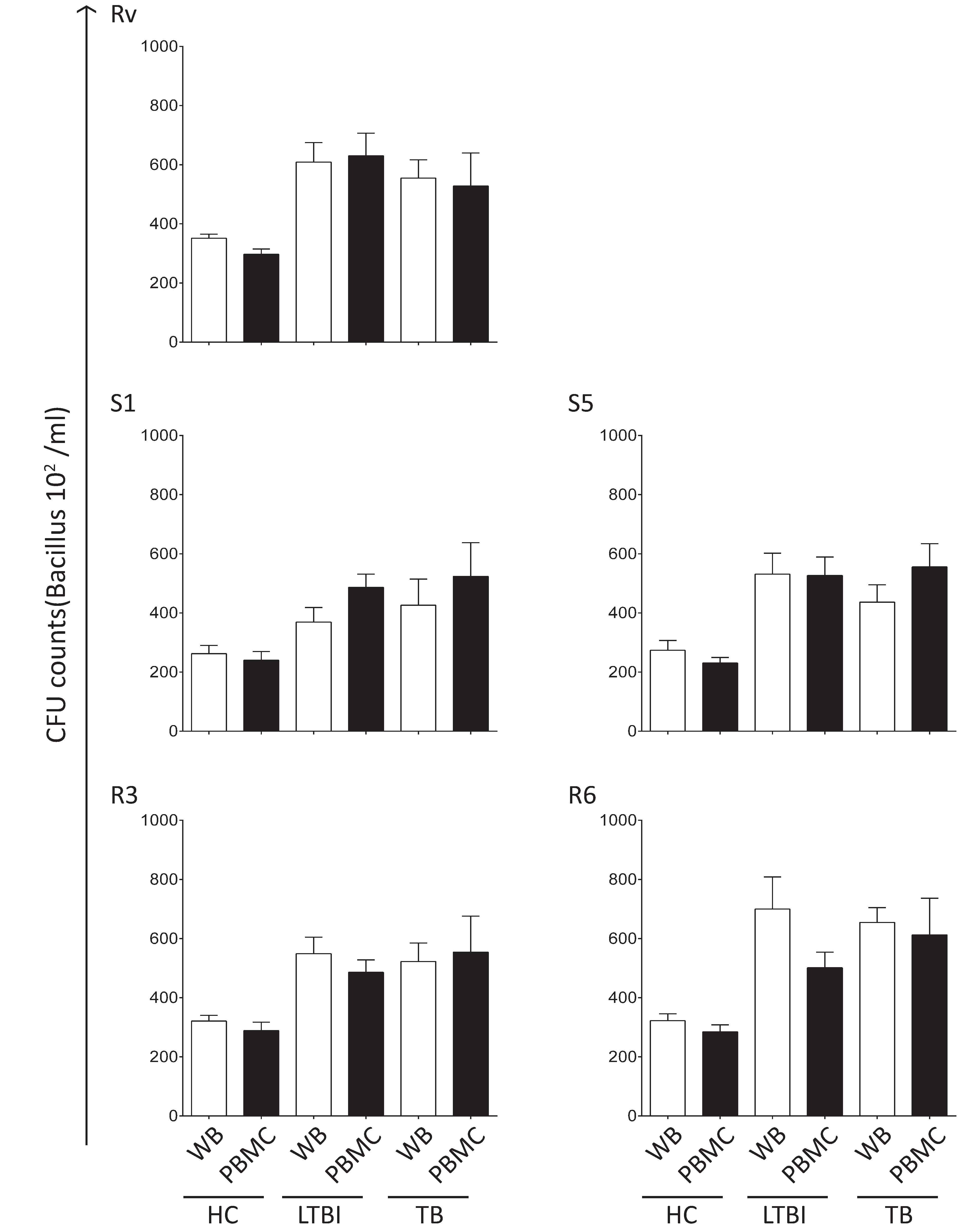

Supplement: S4 Fig — Paired whole blood (WB) and PBMC samples from healthy control subjects (n = 10), LTBI individuals (n = 10) and TB patients (n = 7) were infected with the reference strain H37RV or drug-susceptible (S1 and S5) or multidrug-resistant (R3 and R6) clinical isolates. (TIF) [file pntd.0009605.s004.tif]
